# Experimental study of fat derived pellets promoting wound healing in rats

**DOI:** 10.1080/21655979.2021.2000257

**Published:** 2021-12-11

**Authors:** Li Zeng, Shaolong Zhou, Chen Chen, Lin-Hua Zhou, Xiujiang Shi, Zongjian Wu, Sheng-Kang Luo

**Affiliations:** a Department of Plastic and Reconstructive Surgery, Guangdong Second Provincial General Hospital, Guangzhou City,China; b Yichun University, Yichun, Jiangxi, China

**Keywords:** Stem cell, physical method, macrophages, growth factors, wound healing

## Abstract

To observe the effect of fat-derived pellets (FDP) on wound healing in rats, the inguinal fat of rats was obtained, and the FDP were obtained after centrifugation. The cell activity and growth factor secretion of FDP were measured. The wounds in rats were created, and FDP was used to treat the wounds of rats. The phenotype of macrophages and the expression of angiogenic factors expression in wounds were measured. The cell viability in FDP remains in high level after centrifugation and the expression of vascular endothelial growth factor (VEGF) and Basic Fibroblast Growth Factor (bFGF) from FDP was observed in vitro. The FDP significantly promoted the wound healing of rats compared with that in control groups. Moreover, the expression of M2 macrophages and VEGF in FDP group were significantly higher than that in the control group. FDP is a kind of stem cell product, which can be obtained from adipose tissue by physical centrifugation. The cytotherapeutic effect of FDP makes it a promising product for wound healing in clinics.

## 1. Introduction

Skin defects can be caused by trauma, burns and radiation. Complications such as infection can occur when the body loses the skin barrier [1]. At present, there are many methods for the treatment of skin defects, but many of them are not satisfactory [2]. Stem cells, which secrete various growth factors and angiogenic components, have been considered as an ideal way to treat wounds in recent years [1–3]. Adipose tissue is a natural stem cell pool. Adipose tissue interstitium contains abundant components of stromal vascular fraction, and stromal vascular fraction (SVF) cells contain a large number of adipose-derived stem cells [4,5]. Currently, the most common way to obtain Adipose Derived Stem Cells (ADSCs) is digestion of adipose tissue by collagenase [6]. However, the acquisition process requires the introduction of exogenous enzyme proteins and specialized laboratory equipment and techniques. Therefore, its safety and economy are the two major issues that limit the clinical application of adipose stem cell therapy. Therefore, if ADSCs can be quickly obtained by physical means and applied to wound treatment, it will bring new ideas for the application of stem cell therapy. In the process of treating adipose tissue, our research group found that after centrifugation, more tissue particles would precipitate at the bottom of the obtained adipose tissue. The study found that the tissue granule component contained fat-derived stem cells [7], we speculate that this tissue granule component can be used for wound treatment. To verify this conjecture, we collected the tissue granule components and detected the content and function of ADSCs in it. In addition, a rat wound model was established, tissue granule components were used to treat the wound, and the content and expression of epidermal growth factor and other proteins in the wound were detected to explore its ability to promote wound healing in rats.

## 2. Materials and Methods

### Experimental animals

Twenty-four male Sprague-Dawley rats (License No.: SCXK 2009–0013) were purchased from Changsha Tianqin Biotechnology Co., Ltd., 6 ~ 8 weeks of age. They were raised in a single cage with free food and water. The experiment was carried out after being approved by the Animal Ethics Committee of Yichun University (approval number: 2,019,026).

### 2.1 Fat preparation and FDP acquisition

**Figure 1.**
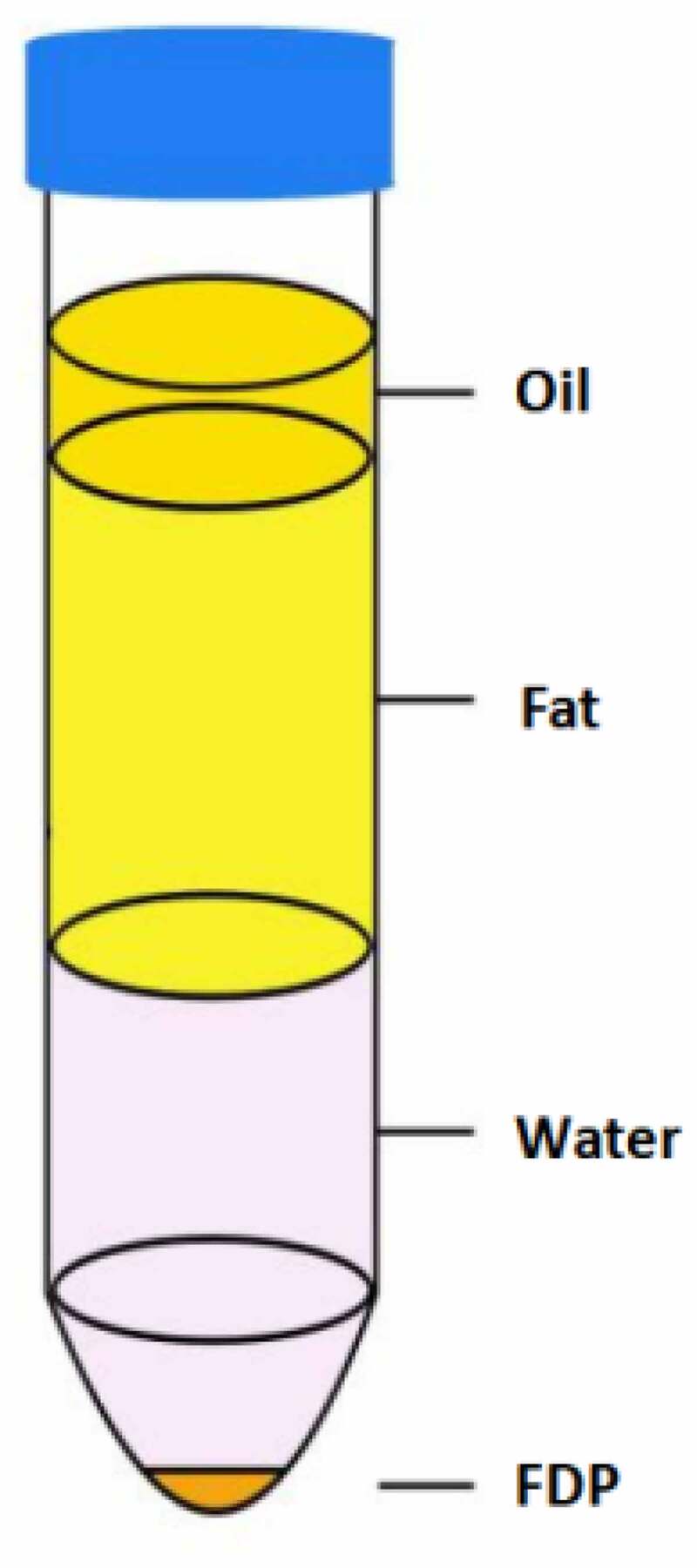
Fat Tissue Derived Pellets (FDP)

The rats were anesthetized with pentobarbital sodium, the abdominal hair was shaved and the abdominal skin was disinfected, and the skin in the groin was cut with sterile instruments. The groin fat was carefully separated and collected. The collected fat was carefully cut into pieces with scissors, and the fascial components were removed. The cut adipose tissue was mixed with normal saline in equal volume and centrifuged at 1200 g for 3 min. The tissue was stratified after centrifugation. From top to bottom, they are oil layer, fat layer, water layer and tissue granular layer. We named the tissue granular layer as Fat Tissue Derived Pellets (FDP) (Figure 1).

### 2.2 Cell activity and growth factor secretion

The obtained FDP components were resuspended in 1 mL of normal saline, and cell apoptosis was detected by flow cytometry using 7-AAD (00–6993-50, Thermo Fisher Scientific, USA) as an indicator of apoptosis. The obtained FDP was resuspended in a 24-well plate, and the medium (DMEM containing 10% (v/v) fetal bovine serum, 100 U/mL penicillin (Gibco BRL), and 100 mg/mL streptocin (Gibco BRL)) was added into the well plate for culture for 48 hours. In order to detect the paracrine function of stem cells in tissue granules, the medium was replaced with DMEM medium without fetal bovine serum for another 24 hours. The content of Vascular growth factor (VGF) in the culture medium was determined by ELISA kit after filtration. Vascular endothelial growth factor (VEGF) (RAB0511, Sigma-Aldrich); fibroblast growth factor, (FGF) (RAB0184, Sigma-Aldrich). MSC identification was performed on the obtained FDA to determine whether the extracted cell components were successful

### 2.3 Rat wound model

The rats were anesthetized with pentobarbital sodium, and a round full-thickness wound with a radius of 1.5 cm was made dorsal. The 24 rats were divided into a control group and an FDP group. The wounds in the control group were treated with 0.2 ml normal saline injection. The wound of the FDP group was treated with 0.2 ml FDP suspension containing 1 × 105 cells. After treatment, the wound was bandaged with sterile dressing and replaced every other day. Skin wound photos were taken at 1, 14 and 21 days after surgery, and the remaining wound area was quantified by ImageJ software.

### 2.4 Immunohistochemistry and gene expression assessment

The skin wound tissues were collected under anesthesia at days 1, 14 and 21. The wound samples were cut in half, and half were fixed, embedded and sected for immunofluorescence staining of macrophages. Anti-rat macrophage markers CD68 (125,212, Abcam, Cambridge, Massachusetts, USA) and CD206 (ab64693, Abcam, Cambridge, Massachusetts, USA) were used for immunofluorescence staining of skin samples, and 4ʹ,6-diamidino-2-phenylindole (DAPI) was used for contrast staining of nuclei. The other half were tested for the expression of the angiogenic gene (VEGF).

### 2.5 FGF-1 level was detected by Western-blot

The collected wound tissue samples were placed in a tissue homogenizer, and the precooled RIPA lysate and protease inhibitor were added. The tissue homogenization was carried out with an ultrasonic crushing machine. The centrifugation was conducted at 12000 rpm at 4°C for 20 minutes, and the supernatant was taken for use. BCA method is used to measure the protein concentration, and the volume of the samples with the measured concentration is calculated by the mass first. Since the volume of each sample is different, RIPA is added to the same volume, so that the volume and concentration of the samples are the same, and then equal volume SDS is added to the same volume. 2× SDS sample buffer was mixed with the protein sample in the same volume, and the protein was denaturated at 95°C for 5 min to ice bath for 10 min, and stored at −20°C for later use. SDS-PAGE (polyacrylamide) gel electrophoresis isolates the protein. 20 μg protein sample was taken, 10% SDS-PAGE electrophoresis, transferred to nitrocellulose film at 100 V for 1 h, and then placed in the blocking solution at 37°C for 1 h. The rabbit FGF-1 Antibody (AmyJet Scientific, 1:2000) was 4°C overnight. Wash with TBST for 3 × 15 min and add secondary antibody (1:1000/1:2000) for 1 h at room temperature. Wash with TBST for 3 × 15 min. Western blotting was used for observation and the absorbance (A) of each band was determined by image analysis for quantitative analysis.

### 2.6 ELISA was used to detect the level of wound collagenase-1

The collected wound tissue samples were placed in a tissue homogenizer, and the precooled RIPA lysate and protease inhibitor were added. The tissue homogenization was carried out with an ultrasonic crushing machine. The centrifugation was conducted at 12000 rpm at 4°C for 20 minutes, and the supernatant was taken for use. Rat collagenase-1 kit (Baiye Biotechnology Co, Shanghai) and double antibody sandwich ABC-ELISA method were used to determine the level of wound collagenase-1: After the well plate was balanced at room temperature for 20 min, different concentrations of collagenase-1 standard were added to the standard well, the sample well was added to the test sample, and the blank well was left untreated. Add HRP-labeled antibodies to the standard well and the template well, cover the template, and let stand at room temperature for 30 minutes. Discard the solution in the hole and wash with washing liquid. Blot dry with absorbent paper. Add substrate solution to each well and leave in darkness for 30 min. Add 0.05 ml of termination solution to terminate the reaction. OD450 was determined with a microplate analyzer.

### 2.7 Statistical analysis

SPSS20.0 software was used for statistical analysis, and the measurement data were expressed as the mean standard deviation. Image J software processing system was used to analyze the WB result bands and optical density values. One-way analysis of variance (ANOVA) test was used for comparison between multiple groups. p < 0.05 indicates that the difference is statistically significant.

## 3. Experimental results

### 3.1 Results of cell activity and growth factor secretion in FDP

**Figure 2.**
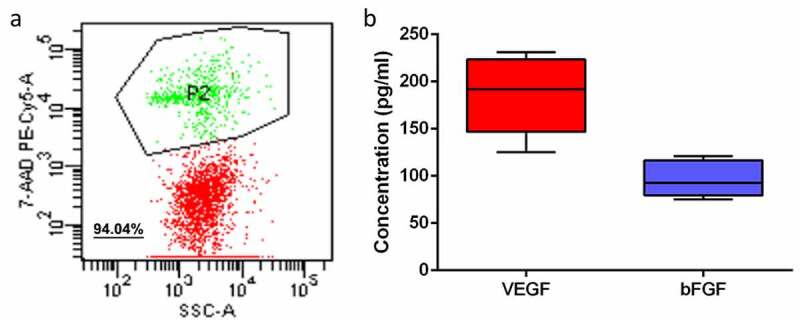
(a): Cell activity in FDP. (b): Results of FDP secreting VEGF and bFGF in vitro

FDP cells obtained from adipose tissue were detected. Flow results showed that the cell activity of FDP obtained by adipose tissue centrifugation remained at a high level (Figure 2a), with an average value of 94.04%. In vitro culture experiments confirmed that FDP cells can secrete VEGF and bFGF, two important angiogenic growth factors. The content of VEGF and bFGF was about 185.8 ± 40 pg/ml and 96.2 ± 19 pg/ml, respectively (Figure 2b).

### 3.2 MSC identification of FDA cells

**Figure 3.**
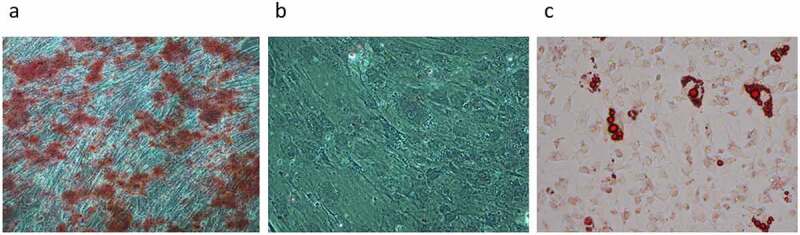
(a): Alizarin red staining for osteogenic differentiated cells. (b): Toluidine blue staining for chondroblast differentiation cells. (c): Oil red O staining for adipogenic differentiated cells

MSC identification was performed on the extracted FDA cells to determine its cell composition. As shown in Figure 3, the result of osteoblasts stained with alizarin red dye showed a large area of purplish red cells (Figure 3a). The result of the toluidine blue staining showed that there were no purplish red chondroblasts in the cells (Figure 3b). Finally, the result of oil-red O staining shows red-yellow fatty oil droplets (Figure 3c). Therefore, the MSC identification results showed that the extracted FDA cells contained a large number of osteogenic differentiated cells as well as adipocytes.

### 3.2 FDP promoted wound healing in rats

**Figure 4.**
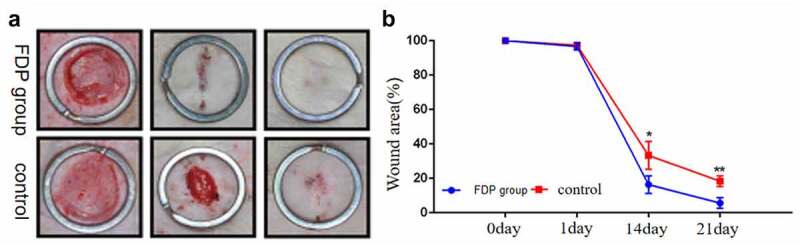
(a):General picture of wound surface of rats (b):Ratio of residual wound area to original wound area. *P < 0.05. **P < 0.01

The gross results showed that there was no statistically significant difference in the wound area between the two groups on the first postoperative day. However, from the 14th day after surgery, the wound area of rats after FDP intervention was significantly smaller than that of the control group. On the 21st day, the wounds in the FDP group were almost healed, and there were still unhealed wounds in the control group (Figure 4).

### 3.3 FDP promoted the expression of M2 macrophages and VEGF in wound skin

**Figure 5.**
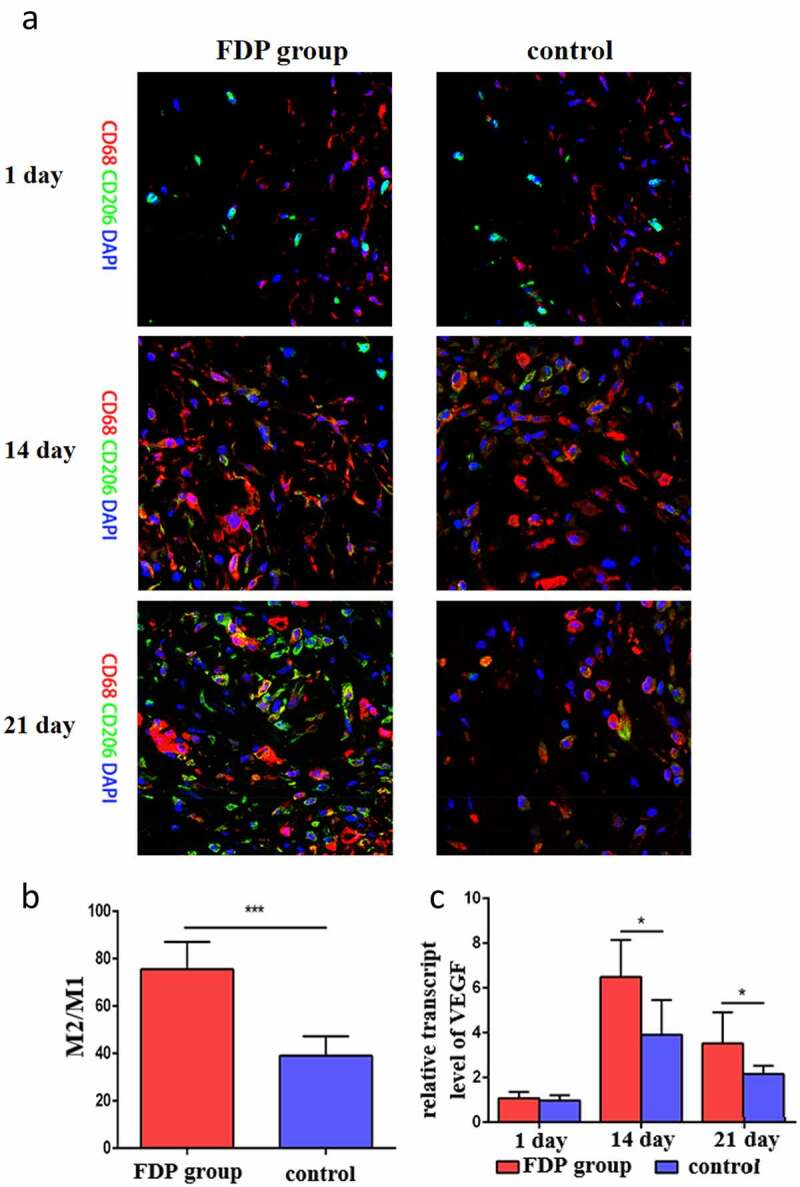
(a): On day 1, day 14, day 21, M1M2 macrophages were stained. (b): Quantitative results of M2/M1 ratio on day 21. (c): VEGF gene expression results. *P < 0.05.***P < 0.001.Scale = 200um

Immunofluorescence staining showed that there was no significant difference in the number of macrophages between the two groups on day 1. On day 14, the number of macrophages in FDP group began to increase, which was significantly higher than that in control group. On day 21, the number of CD206 in FDP group increased significantly, and there were a wide range of M2-type macrophages in the wound skin of the FDP group (Figure 5a), and the quantitative results showed that the proportion of M2-type macrophages was significantly higher than that of the control group (Figure 5b). Meanwhile, the expression of pro-vascular growth factor VEGF in wound skin of FDP group was also significantly higher than that of the control group (Figure 5c).

### 3.4 FDP promoted the expression of FGF-1 and collagenase-1 in wound skin

**Figure 6.**
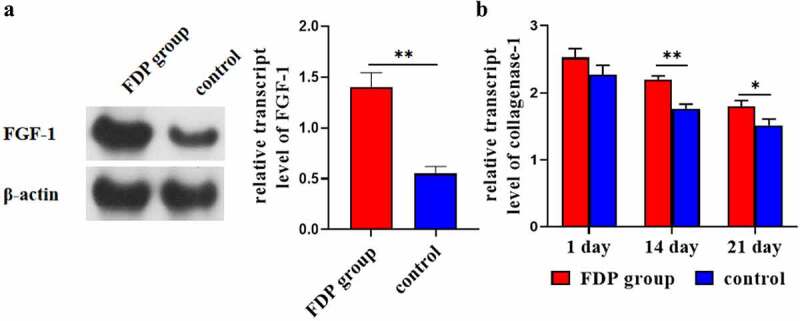
(a) Expression levels of FGF-1 in FDP group and control group. (b) Relative expression level of collagenase 1 in wound skin tissue. *P < 0.05. **P < 0.01

WB results showed that at day 21, the expression of FGF-1 in wound skin of the FDP group was significantly higher than that of the control group (Figure 6a). ELISA results showed that the expression level of collagenase-1 in wound skin in the FDP group was significantly higher than that in the control group at the 14th and 21st day (Figure 6b).

## 4. Discussion

Healing after skin injury is a process of dynamic coordination of various cells, including inflammation, cell proliferation and remodeling [8]. Studies have shown that the dynamic coordination of various cells in the process of wound healing requires the regulation of various growth factors [9,10]. A loss of cells or growth factors in either link leads to slow wound healing. The effectiveness of current wound treatment methods is limited by the above factors, and the cost is high. Therefore, regenerative medicine has become an alternative for wound treatment and wound healing, and can effectively improve wound healing and restore normal skin structure [11–13]. Stem cell therapy is a promising therapeutic approach in regenerative medicine. Stem cells have the ability to self-renew and differentiate to produce multiple cell types, which are essential for wound healing.

Among many stem cells, mesenchymal stem cells (MSCs) and adipogenic stem cells (ADSCs) have attracted much attention as suitable candidates for promoting tissue regeneration [14]. Studies have shown that artificially induced mesenchymal stem cell-derived exosomes can promote collagen synthesis and angiogenesis and promote skin wound healing in rats [15]. However, in MSC-based therapy, implanted MSCs have poor survivability and reduced safety. Several strategies have been developed to improve the survival rate of transplanted mesenchymal stem cells [16]. In addition, the self-renewal ability and molecular mechanism of MSCs remain unclear [17]. Adipogenic stem cells are a type of mesenchymal stem cells, which are widely distributed in the subcutaneous tissue and play an important role in maintaining the structure of skin tissue. ADSCs can secrete abundant secretors, which can facilitate cell proliferation and differentiation, improve cells and protect the microenvironment [18–23]. ADSCs are often found in liposuction, which can promote the survival of adipose tissue after transplantation and improve cell proliferation rate, thus speeding up wound healing [24,25]. However, during the suction process, the activity of ADSCs was decreased due to the absence of the original microenvironment. The transplanted stem cells often undergo apoptosis after a few days [26].

In addition, in 2006, induced pluripotent stem cells (iPSCs) were developed for the first time to address the limited source and ethical constraints of stem cells. IPSCs are induced by transcription factors to return adult stem cells to a state similar to embryonic stem cells with good differentiation potential. IPSCs can be used for autotransplantation to avoid immune rejection and effectively improve the survival rate [27,28]. In order to avoid the short-term apoptosis of transplanted stem cells, electrospun polycaprolactone/gelatin scaffolds can be used to effectively improve the survival time of transplanted stem cells [29,30]. However, the tumorigenic potential of induced pluripotent stem cells should not be ignored. The transcription factors, vectors and undifferentiated induced stem cells may bring tumorigenic risk. To address the risk of tumorigenicity, making lysine-specific demethylase 1 inhibitors unregulated in tumorigenicity cells eliminates undifferentiated induced pluripotent stem cells and prevents tumor formation [31]. Brentuximab vedotin targets CD30 in undifferentiated cells and induces apoptosis. Has been used to eliminate the tumorigenic potential of cardiomyocyte derived iPSCs [32]. However, the safety and economics of stem cell application are still two important factors limiting the extensive clinical application of stem cells.

Stem cell therapy is one of the most attractive projects in the field of regenerative medicine in recent years. At present, most stem cells are obtained through collagenase digestion, which is easy to cause the contamination of heterogeneous enzyme protein and has certain biological risks [33]. Moreover, this process of stem cell separation requires experienced laboratory operators and specialized laboratory equipment and reagents, which increases the cost of stem cell acquisition. In this study, FDP, a component of adipose tissue particles rich in stem cells, was obtained by physical centrifugation. Similar reports have suggested that the adipose tissue granule component contains SVF cells and may also contain various growth factors [7,34]. Therefore, the stem cell product FDP used in this study is obtained by pure physical means, which has certain advantages over previous stem cell products in terms of safety and economy. However, this study has not explored the specific stem cell components in FDP, and the specific components of growth factors secreted by cells in FDP are still not fully understood. Therefore, follow-up studies will focus on these two issues.

In addition, studies have shown that inflammation is closely related to tissue regeneration [35]. Among various inflammatory cells, macrophages are involved in the whole process of tissue healing and play an important role in the regeneration of various tissues [36,37]. Macrophage phenotypes are generally classified as pro-inflammatory M1 and anti-inflammatory M2. In particular, M2 macrophages are generally thought to promote angiogenesis and inhibit inflammation, while M1 macrophages are thought to inhibit angiogenesis and promote inflammation [38]. Therefore, the higher the local M2/M1 ratio, the stronger the signal of angiogenesis and regeneration. In this study, we detected a higher proportion of M2 macrophages and vascular growth factor VEGF expression in the wound skin in the FDP group than in the control group. This suggests that FDP can induce the phenotype transformation of local macrophages to M2 type, and a high proportion of M2 type macrophages is likely to further induce local angiogenesis, thus promoting local wound healing.

FDP is a kind of stem cell product obtained by pure physical way, and it can play a certain function of stem cell therapy, and may become a new choice for clinical treatment of wounds. However, there are still areas to be improved in this subject, such as whether there is a difference in the yield of FDP obtained by different centrifugal forces, and whether different centrifugal forces have an impact on cell viability in FDP.

## 5. Conclusion

In this study, stem cell components (FDP) were obtained from liposuctioned fat by means of physical homogenate, and FDP was applied to the wound surface of rats to observe its effect on wound healing. The results showed that FDP had high cell activity and could release a large number of growth factors through paracrine action. At the same time, in vivo experiments also confirmed that FDP can effectively promote wound healing and induce the expression of wound M2 macrophages and pro-vascular factor VEGF. Fibroblast growth factor FGF-1 can stimulate the proliferation of almost all wound cells. WB results showed that FDP could promote the expression of FGF-1, thus promoting the proliferation of wound cells and accelerating wound healing. ELISA test showed that FDP could promote the expression of collagenase-1 in wound tissue and enhance the healing ability of wound. This shows that it is feasible to isolate stem cells physically and use them in clinical treatment. This method is simple in process and low in cost, which can effectively reduce the cost of treatment, reduce the economic burden for patients, and facilitate the popularization of stem cell therapy.
